# Seroprevalence of hepatitis E virus differs in Dutch and first generation migrant populations in Amsterdam, the Netherlands: a cross-sectional study

**DOI:** 10.1186/s12879-016-2007-z

**Published:** 2016-11-08

**Authors:** S. Sadik, G. G. C. van Rijckevorsel, M. S. van Rooijen, G. J. B. Sonder, S. M. Bruisten

**Affiliations:** 1 Department of Infectious Diseases, Public Health Service of Amsterdam, Nieuwe Achtergracht 100, 1018 WT Amsterdam, The Netherlands; 2 Academic Medical Centre, Division of Infectious Diseases, Amsterdam, The Netherlands; 3 National Institute for Public Health and the Environment, RIVM, Bilthoven, The Netherlands

**Keywords:** Hepatitis E virus, Seroprevalence, Ethnic groups, Migration age, Amsterdam

## Abstract

**Background:**

In the last decade hepatitis E virus (HEV) is increasingly recognized as a cause of acute viral hepatitis in developed countries. HEV is transmitted via the fecal-oral route. In countries like the Netherlands, HEV infection is suspected to be a zoonosis but HEV may also be introduced by migrants. We studied the seroprevalence of HEV among different migrants, mainly Moroccans and Turks, and compared this to that of the native Dutch population in Amsterdam, the Netherlands.

**Methods:**

Data were obtained from a cross-sectional survey of the adult Amsterdam population performed in 2004; the Amsterdam Health Monitor. A total of 1199 plasma samples were tested for IgG-and IgM antibodies to HEV using the Wantai kit according to instructions of the manufacturer. Basic demographic data (gender, age, country of birth, and age at immigration) were used in the analyses. Hepatitis A virus (HAV) serology data were available from a previous study.

**Results:**

The total weighted anti-HEV IgG seroprevalence in the overall Amsterdam population was 26.7 %, based on 1199 samples. In the study population (not-weighted) this HEV seroprevalence was 157/426 (36.9 %) for the Dutch participants and it was 161/257 (62.6 %) for Moroccans, 99/296 (33.4 %) for Turks and 42/220 (19.1 %) for other ethnicities. HEV seroprevalence increased significantly with age. First-generation Moroccan migrants (44.0 %) had a significantly higher weighted HEV seroprevalence than the Dutch participants (29.7 %). In the first generation Turks (20.3 %) and first generation migrants from other countries (16.7 %) this weighted seroprevalence was lower, but this was only significant for the ‘other ethnicities’. The median age of migration was significantly higher in the Moroccan and Turkish migrants who were HEV IgG positive versus HEV IgG negative. However, when stratifying for age at time of study, median migration age was only significantly different for HEV sero-status for younger Turks and younger ‘other ethnicities’. HEV IgM antibodies were found in 0.6 % (*n* = 7) of participants and none were positive for HEV RNA, showing that there were no acute infections. Despite the common route of fecal-oral transmission for both viruses, there was no relation between HEV and HAV seropositivity.

**Conclusion:**

Within the multi-ethnical capital city of Amsterdam the HEV seroprevalence in first generation migrant populations differed from each other and from the autochthonous Dutch population. The relation between being HEV seropositive and a higher median age of migration suggests that younger migrants got more often infected in their country of origin than in the Netherlands.

## Background

Since 1980, hepatitis E virus (HEV) is recognized as a causative agent of acute viral inflammation of the human liver [1]. The clinical features resemble those of infection with hepatitis A virus (HAV), and both are transmitted by the fecal-oral route or by contaminated water [2]. Unlike HAV, secondary transmission appears to be of minor significance [3, 4]. HEV infection is usually self-limited, and its severity may range from subclinical infection to fulminant liver failure, probably depending on genotype. The risk for a fulminant disease course is particularly high in pregnant women, with an increased risk for adverse pregnancy outcomes and a maternal mortality rate up to 20 % in case of a genotype 1 infection [5–7]. In addition, an increased risk for complications is observed in immunosuppressed persons, especially in transplant patients who may develop chronic hepatitis [8, 9].

HEV, a non-enveloped single-stranded RNA virus, is classified as a unique member of genus *Hepevirus* in the family of *Hepeviridiae*, of which only one serotype is known [10]. Four human HEV genotypes have been identified, each having distinct geographical distributions. Genotypes 1 and 2 are responsible for large water-borne HEV outbreaks in humans in tropical and sub-tropical parts of Asia, Africa and Central America due to poor hygiene. In these areas HEV infection is considered an important cause of acute clinical hepatitis in adults [11, 12]. In developed Western countries HEV genotypes 3 and 4 are thought to be zoonotic as they are found in domestic pigs and cows and also in wild animals such as swine, deer, and mongoose [13]. The exact route of transmission of genotypes 3 and 4 is still unknown, but it may be by eating contaminated undercooked food such as meat from domestic pigs, or from wild animals [12–15].

In the Netherlands, the incidence of HEV is unknown, and representative data on the seroprevalence of HEV antibodies in the general Dutch population are scarce. In 2011 it was estimated that on average 27 % of Dutch blood donors were anti-HEV IgG positive, with an age-dependent increase from 13 % in teenagers to 42 % in those of 50 years and older [16].

This study investigated a representative sample of the general adult population of Amsterdam, the Netherlands, on the presence of antibodies against HEV in relation to demographic data. Amsterdam has large migrant communities, mainly from Morocco and Turkey, but also from people originating from various tropical countries such as Surinam, Netherlands Antilles and Indonesia. This allowed us to look for determinants for anti-HEV seropositivity in different migrant groups using data and samples from a study that was performed in 2004, the Amsterdam Health Monitor (AHM). Furthermore, we looked whether anti-HEV seropositivity was related to the seroprevalence of hepatitis A virus (HAV) in the same AHM population [17]. The resulting data offers new insights into the sero-epidemiology of HEV infection in an urbanized area with mixed ethnicities.

## Methods

### Study population and sampling procedure

The data used for this study were obtained from a cross-sectional survey, the Amsterdam Health Monitor (AHM) study, carried out in 2004 by the Public Health Service of Amsterdam (GGD Amsterdam) and was approved by the Medical Ethics Committee of the Academic Medical Centre in Amsterdam, the Netherlands. A random sample of residents aged 18 years or older was selected from the municipal register in Amsterdam. The sample was stratified by age and ethnicity, with oversampling of Turkish and Moroccan persons. After providing written informed consent, participants were interviewed and gave blood samples. Within 48 h the plasma samples were stored at −80 °C. Of those interviewed, 79 % (*n* = 1376) provided blood samples of whom 1294 (94 %) plasma samples were available for HEV antibody testing. Of these, 1199 (93 %) samples from Dutch participants and first generation migrants were included in the statistical analyses. Further details of the AHM study population are described by Agyemang et al., 2006, and in other studies [17–19].

For this study, the participants were classified into four ethnic groups: Dutch, Turkish, Moroccan, and ‘other’, according to the self-reported country of birth of the participant’s mother. If the mother was born in the Netherlands or country of birth was unknown, the self-reported country of birth of the participant’s father was used. All participants of non-Dutch ethnic origin were classified as first (born outside the Netherlands)- or second (born in the Netherlands)-generation migrants. The second generation participants were excluded in this study, because of small numbers (*n* = 87) and the fact that their ethnicities were very diverse.

### Laboratory testing

Plasma samples from participants were tested for antibodies to HEV (anti-HEV IgG, anti-HEV IgM) by means of an enzyme immunoassay according to instructions of the manufacturer (Wantai Biological Pharmacy Enterprise Co., Ltd , Beijing, China). Antibodies to HAV were tested as described elsewhere [17].

Reverse transcriptase PCR was performed to detect HEV RNA according to a previously published protocol [16].

### Statistical analysis

To make the results representative for the adult population in Amsterdam, prevalences and 95 % confidence intervals (95 % CI), were calculated using the weighted population fraction and complex samples module of SPSS. The data were weighted for age, sex, and ethnic origin, by correcting for the oversampling by ethnic groups as previously described [18]. Prevalences were calculated and compared using Chi-squared test. Univariable and multivariable logistic regression with backward selection method were used to test for the association between demographic covariates and HEV seropositivity. Differences between HEV IgG seropositive and seronegative groups with respect to median age of migration was calculated per ethnicity using the Mann–Whitney U test. Values of *p* < 0.05 were considered significant. Calculations were performed in SPSS, version 20 (SPSS Inc., Chicago, Illinois, USA).

## Results

### Characteristics of the study sample

The study sample of 1199 participants consisted of 558 men (46.5 %) and 641 women (53.5 %) (Table 1). Age ranged from 18 to 90 years. The median age for men was 51 years (IQR 19 years) and for women 49 years (IQR 20 years). Most participants were Dutch (35.5 %) and almost all 753 first generation migrant participants came from Turkey (296; 24.7 %) or Morocco (257; 21.4 %), due to deliberate oversampling among Turkish and Moroccan populations. Most ‘others’ came from the Republic of Surinam (*n* = 60) or the Netherlands Antilles (*n* = 11). Participants were equally distributed over the sexes, except for Moroccans, of whom 58.4 % was male, and for Dutch participants, of whom 58.9 % were women. The median age at migration was 25 years (range 0–76 years), and only a minority (8 %) migrated to the Netherlands before the age of 15 years.

**Table 1:** Prevalence of anti- HEV IgG in association with demographic characteristics in the adult Amsterdam population in 2004

Characteristic	Study sample									Amsterdam adult population	
	Total tested	HEV- seropositive		Univariate OR (95 % CI)			Multivariate OR^a^ (95 % CI)			Weighted HEV seropositive^a^	95 % CI
		No.	(%)			*P* value			*P* value	%	
Total	1199	459	(38.3)							26.7	(23.8–29.8)
Gender						0.002			0.54		
Male	558	239	(42.8)	1						26.0	(22.0–31.1)
Female	641	220	(34.3)	0.7	(0.6–0.9)					27.0	(23.2–31.1)
Age category						<0.001			<0.001		
18–34	162	15	(9.3)	1			1			9.8	(5.5–17.0)
35–44	269	64	(23.8)	3.1	(1.7–5.6)		3.0	(1.6–5.5)		20.7	(15.1–27.7)
45–54	308	124	(40.3)	6.6	(3.7–11.8)		7.5	(4.2–13.7)		33.3	(27.2–40.1)
55–64	257	135	(52.5)	10.8	(6.0–19.5)		11.7	(6.4–21.5)		42.7	(35.3–50.4)
65 and older	203	121	(59.6)	14.5	(7.9–26.4)		18.1	(9.7–33.8)		54.4	(45.8–62.8)
Ethnic origin						<0.001			<0.001		
Dutch	426	157	(36.9)	1			1			29.7	(25.7–34.0)
Moroccan	257	161	(62.6)	2.9	(2.1–4.0)		3.5	(2.4–4.9)		44.0	(37.1–51.1)
Turkish	296	99	(33.4)	0.9	(0.6–1.2)		1.1	(0.8–1.5)		20.3	(17.0–24.0)
Other^b^	220	42	(19.1)	0.4	(0.3–0.6)		0.4	(0.3–0.6)		16.9	(12.0–23.3)

^a^The weighted seroprevalence was calculated, taking fractions of ethnical residents in Amsterdam into account, as explained in Methods

^b^Other countries are mainly Surinam, Netherlands Antilles and Indonesia

### Seroprevalence and determinants of anti-HEV in the Amsterdam population

Seven of the 1199 available plasma samples (0.6 %) tested positive for anti-HEV IgM, and none of them were HEV RNA positive. This indicates that there were no or very few acute HEV infections. The 7 samples were from 4 Dutch participants, one Moroccan, one Turkish, and one participant from former Yugoslavia.

Of 1199 plasma samples 459 (38.2 %) tested positive for anti-HEV IgG. The HEV seroprevalence is shown in Table 1, both non-weighted, representing the study sample, and weighted, representing the Amsterdam population. The weighted anti-HEV IgG seroprevalence in the total Amsterdam population in the year 2004 was 26.7 % (95 % CI 23.8–29.8 %). The weighed anti-HEV IgG seroprevalence increased from 9.8 % in the youngest age category (18 to 34 years) to 54.4 % in the oldest age group (older than 65 years).

In univariable analysis, anti-HEV IgG was associated with gender, age and ethnic origin (Table 1). Women were significantly less likely to be anti-HEV positive compared to men. In multivariable analysis, age and ethnic origin remained independent predictors for anti-HEV IgG seropositivity.

The weighted seroprevalence was highest among first-generation Moroccan migrants (44.0 %) and they were significantly more often anti-HEV IgG positive compared to those of Dutch ethnic origin with a seroprevalence of 29.7 % (OR 3.5; 95 % CI 2.4–4.9). The weighted seroprevalence of anti-HEV IgG in first-generation Turkish migrants was 20.3 % which did not differ significantly from the Dutch (OR 1.1; 95 % CI 0.8–1.5). From ‘other’ countries the weighted HEV seroprevalence was 16.9 % which was in multivariate analysis significantly lower than the seroprevalence in the Dutch population (Table 1).

We examined the relation between age of migration and being seropositive for HEV. Significant differences were seen for both the Moroccan and Turkish groups, with a higher median migration age of those who were HEV IgG positive versus HEV IgG negative (Table 2). Since HEV positivity was found to increase with age, we also compared the median ages of migration in stratified groups per ethnicity. The stratification was made with the median age of participation as cut-off. The differences were no longer significant for the Moroccan groups, with comparable ages of migration in the ‘younger’ and ‘older’ participants (Table 2). For the Turks however, the median age of migration in the ‘younger’ group was significantly higher in the HEV IgG positive group (24 years) than in the HEV IgG negative group (18 years). Also in the ‘other ethnicities’ the median age of migration in the younger group was significantly higher in the HEV IgG positive (27 years) versus the HEV IgG negative (23 years) group (Table 2). This indicates in ‘younger’ immigrant (except Moroccans) that those who migrated at a younger age to the Netherlands were less likely to be HEV infected.

**Table 2:** Relation between hepatitis E virus IgG and median age of migration per ethnicity of first generation migrants

Ethnicity	Stratification by median age (years)^a^	HEV IgG Negative		HEV IgG positive			*P* value^d^
		N	Median age of migration^b^ (SD)	N	Median age of migration^b^ (SD)	Missing^c^ N	
Moroccan	none	95	26 (11.0)	154	27 (9.4)	8	**0.021**
	≤51	68	21 (11.7)	55	23 (8.7)	3	0.310
	>51	27	29 (6.0)	99	29 (8.7)	5	0.493
Turkish	none	195	23 (9.5)	97	29 (8.5)	4	**<0.001**
	≤46	122	18 (7.7)	22	24 (7.6)	1	**0.003**
	>46	73	29 (8.3)	75	30 (8.1)	3	0.321
Other	none	173	25 (13.3)	38	27 (10.1)	9	0.367
	≤50	82	23 (9.7)	18	27 (7.7)	4	**0.028**
	>50	91	28 (14.9)	20	27 (12.1)	5	0.466

Differences in median migration ages between HEV IgG negative and HEV IgG positive groups were tested using the Mann–Whitney *U* test

^a^Median age of participants at the time of the study

^b^Median age at which participants migrated to the Netherlands

^c^Missing: no data available on age of migration

^d^Numbers in bold indicate significant differences

### Anti-HAV status

The HAV IgG status was known from a previous study [17] and was available for all 1199 samples. Those who were HAV IgG positive were compared for two subgroups: participants who were anti-HEV IgG positive and anti-HEV IgG negative (Table 3). Of the 459 anti-HEV seropositive persons 374 (81.5 %) had also antibodies for HAV. This was not significantly different in the anti-HEV IgG seronegative group (73.1 %). For the Dutch participants with 48.4 % HAV IgG seropositive there was also no significant difference in HAV status in relation to anti-HEV seropositivity. Almost all first generation migrants from Morocco and Turkey (97.7 to 99.3 %) were HAV seropositive, regardless of their anti-HEV IgG status (Table 3). For the ‘other ethnicities’ there were also no significant differences between the two groups for anti-HEV IgG status. This indicates that there is no association between infection with HEV and infection with HAV in these populations.

**Table 3:** Relation between hepatitis E virus IgG and hepatitis A virus IgG seropositivity in Dutch and first generation migrants of the Amsterdam health monitor study

	Anti-HEV IgG positive			Anti-HEV IgG negative			Total		
Ethnicity	N^a^	HAV IgG positive	%	N^a^	HAV IgG positive	%	N^a^	HAV IgG positive	%
Dutch	157	83	52.9	269	123	45.7	426	206	48.4
Moroccan	161	160	99.4	96	91	94.8	257	251	97.7
Turks	99	99	100	197	195	99.0	296	294	99.3
Other	42	32	76.2	178	147	82.6	220	179	81.4
Total	459	374	81.5	740	556	73.1	1199	930	77.6

^a^ *N* number included

## Discussion

In this cross-sectional study among the adult population of Amsterdam, we found clear differences in HEV seroprevalence between those born in the Netherlands and first generation migrants. This has not been reported before within one multi-ethnic population. Also new in this study is that weighted calculations could be made in the AHM study. The overall weighted seroprevalence of HEV-IgG in the adult Amsterdam population was 26.7 % in 2004, which is comparable to an estimate made in 2011 among Dutch blood donors (27 %) by Slot et al. [16]. Yet the blood donor population likely reflects a more autochthonous Dutch population with certainly less Moroccans and Turkish participants, as in the AHM study a deliberate oversampling on these ethnicities was performed. Similar to the blood donor population our study demonstrated an age related increase of anti-HEV seroprevalence of 10 % in those younger than 35 years, to 55 % in persons of 65 years and older. This association of HEV-IgG seropositivity with increasing age has also been reported in other studies [20–23]. In the Netherlands, the anti-HEV seroprevalence in blood donor populations declined over time from 46.6 % in 1988 to 20.9 % in 2011 among comparable age groups, indicating that the HEV infection pressure is not constant. Indeed, in the Netherlands a recent rise of anti-HEV IgG prevalence among young blood donors indicates that the HEV incidence is increasing again [21].

First generation migrants of Moroccan ethnic origin were 3 times more likely to be anti-HEV-seropositive compared to the autochthonous Dutch population. A recent review reported that the anti-HEV seroprevalence in the general population in countries of the Middle East and the Northern African-region, where also Morocco is situated, ranges from 2 to 38 % [24]. This is lower than the 44 % weighted prevalence that we found among first generation Moroccans in Amsterdam.

There was a significant difference in age of migration between those who were HEV positive versus HEV negative in the ‘younger’ Turkish and ‘other’ ethnicity migrants, but not for the Moroccans. This could indicate that those who migrated at an older age from Turkey or the other countries had got infected in the country of origin. Those who were older than 40 to 45 years at the time of study participation had been at least 20 years in the Netherlands (Table 2) and had experienced an equal infection pressure as the Dutch, as shown by the same chance of being HEV positive or HEV negative.

Surprisingly, first generation migrants from Turkey had a lower weighted seroprevalence compared to the Dutch, although this difference was not significant; yet it was also significantly lower than that of first generation Moroccans. This difference is not easily understood. It indicates that exposure to HEV in Turkey differs from that in Morocco, however it might also be that the higher prevalence in Moroccans relative to that of Turkish first generation migrants in Amsterdam reflects a more rural versus a more urban descent of these migrants, respectively. The seroprevalence (20.3 %) that we found in the Turkish group is higher compared to the seroprevalence estimated in a Turkish study (of 2002) showing a geographical range of 3.8 % in the city of Ankara up to more than 15 % in rural Eastern Turkey [25]. Because the assays that were used differ, study results are not fully comparable and the Turkish study possibly underestimated the true prevalence.

Other sero-epidemiological studies in developed countries reported a wide variety of anti-HEV IgG seroprevalence, from 5 % in Japan, to 22 % in France, Germany, and Denmark and also in the United States [20, 22, 23, 26, 27]. Next to differences in exposure to HEV, an important issue is also the variation in assays with different performances that were used [28–31]. In this study the Wantai HEV IgG test was used, with a high specificity (99 %) and sensitivity (98 %) [16, 28, 29, 31–33]. Using this assay the anti-HEV seroprevalence in many developed countries was higher than previously anticipated [11, 25, 34].

A limitation of our study is that HEV genotypes are not known. HEV seropositivity in first generation Moroccans may be based on immunity against HEV genotypes 1 or 2, since these types predominantly circulate in African countries. Also water-borne HEV outbreaks with these genotypes have been described in several parts of Morocco [3, 12, 35]. It might thus well be that first generation Moroccan participants experienced a HEV infection before they migrated to the Netherlands, whereas others got infected in the Netherlands. Due to small numbers, we were only able to create two age strata in the analysis of the median age of immigration. Possibly, residual confounding of age may have influenced our findings.

In the Netherlands it was previously shown that both humans and pigs harbor the same HEV genotype 3, so transmission is probably food-related [14, 15]. Because Muslims do not eat pork meat or other pork products and because Surinamese people are known not liking to eat raw or undercooked meat, these groups may be protected from acquiring HEV genotype 3 in the Netherlands.

We hypothesized that because HAV and HEV are both fecal-orally transmitted infections, there might have been a relation between the seroprevalence of HEV and HAV. Almost all first-generation migrants from Morocco and Turkey had natural immunity to HAV, regardless of their HEV status. Also within the Dutch population there was no difference in HAV status between the anti-HEV IgG positive and negative groups. Our data showed thus no such relation between hepatitis E and hepatitis A virus infection and therefore we think that the transmission of both hepatitis viruses occurred independently. This was also previously shown in many other studies. In most of the countries also the HAV seroprevalence was higher than the HEV seroprevalence, in similar age groups [36–40].

## Conclusions

In conclusion, the differences in HEV seroprevalence in people with different ethnic origins may be associated with a different risk of exposure to HEV in either the country of origin (migration at a younger age) or in the Netherlands (migration at older age). More research is needed on first and second generation migrants to unravel possible cultural and food influences.

## Abbreviations

95 % CI: 95 % confidence interval
AHM: Amsterdam health monitor study
HAV: Hepatitis A virus
HEV: Hepatitis E virus
IgG: Immunoglobulin G
IgM: Immunoglobulin M
IQR: Interquartile range
OR: Odds ratio
